# Effectiveness of osteopathic craniosacral techniques: a meta-analysis

**DOI:** 10.3389/fmed.2024.1452465

**Published:** 2024-10-03

**Authors:** Alfred Amendolara, Alexander Sheppert, Ryan Powers, Andrew Payne, Stephen Stacey, David Sant

**Affiliations:** ^1^Department of Biomedical Science, Noorda College of Osteopathic Medicine, Provo, UT, United States; ^2^Federated Department of Biological Science, New Jersey Institute of Technology, Newark, NJ, United States; ^3^La Crosse-Mayo Family Medicine Residency Program, Mayo Clinic Health System, La Crosse, WI, United States

**Keywords:** osteopathic medicine, craniosacral, osteopathic manipulation (osteopathic manipulative treatment/osteopathic manual medicine), manual therapy, alternative and complementary medicine

## Abstract

**Background:**

Craniosacral osteopathic manipulative medicine—also known as craniosacral therapy (CST)—is a widely taught and used component of osteopathic medicine. This paper seeks to systematically review and conduct a meta-analysis of randomized controlled trials assessing the clinical effectiveness of CST compared to standard care, sham treatment, or no treatment in adults and children.

**Methods:**

A search of Embase, PubMed, and Scopus was conducted on 10/29/2023 and updated on 5/8/2024. There was no restriction placed on the date of publication. A Google Scholar search was conducted to capture grey literature. Backward citation searching was also implemented. All randomized controlled trials employing CST for any clinical outcome were included. Studies not available in English as well as studies that did not report adequate data were excluded. Multiple reviewers were used to assess for inclusions, disagreements were settled by consensus. PRISMA guidelines were followed in the reporting of this meta-analysis. Cochrane’s Risk of Bias 2 tool was used to assess for risk of bias. All data were extracted by multiple independent observers. Effect sizes were calculated using a Hedge’s G value (standardized mean difference) and aggregated using random effects models. The GRADE system was used to assess quality of evidence.

**Results:**

The primary study outcome was the effectiveness of CST for selected outcomes as applied to non-healthy adults or children and measured by standardized mean difference effect size. Twenty-four RCTs were included in the final meta-analysis with a total of 1,613 participants. When subgroup analyses were performed by primary outcome only, no significant effects were found. When secondary outcomes were included in subgroup analyses, results showed that only *Neonate health, structure* (*g* = 0.66, *95% CI* [0.30; 1.02], *Prediction Interval* [−0.73; 2.05]) and *Pain, chronic somatic* (*g* = 0.34, *95% CI* [0.18; 0.50], *Prediction Interval* [−0.41; 1.09]) show reliable, statistically significant effect. However, these should not be interpreted as positive results as wide prediction intervals, high bias, and statistical limitations temper the real-world implications of this finding.

**Conclusions and relevance:**

CST demonstrated no significant effects in this meta-analysis, indicating a lack of usefulness in patient care for any of the studied indications.

Pre-registration available at https://doi.org/10.17605/OSF.IO/54K6G.

**Systematic Review Registration:**

https://osf.io/54k6g.

## 1 Background

Craniosacral osteopathic manipulative medicine—also known as craniosacral therapy (CST) or osteopathy in the cranial field—was developed by William G. Sutherland, D.O. in the 1940s (1, 2). He proposed the existence of an inherent movement between the dura, sacrum, and cranial bones known as the cranial rhythmic impulse (CRI). The basis of this theory is predicated on an inherent rhythmic motion of the brain and spinal cord, flow of cerebral spinal fluid, mobility of cranial and spinal membranes, mobility of the cranial bones at their sutures, and physical connection between the sacrum and the bones and membranes of the cranium (2, 3). Practitioners of CST seek to influence the health of patients through the use of manual techniques to affect the CRI. Craniosacral therapy is currently considered a form of complementary medicine by the World Health Organization (4).

The existence of the CRI is in direct conflict with current understanding of anatomy and development. It has been well established by current and past literature that cranial sutures are fused by early adulthood and have minimal clinically significant motion thereafter (5–11). Thus, despite the theories put forth by proponents of CST, no plausible biological mechanism for CST exists. The use of CST in infants may be less impacted by this, however, due to the increased malleability of the cranium before sutures become ossified. It should also be noted that, with the exception of certain neonatal conditions in which the cranial bones may be manipulated, there is no clear link between the proposed mechanisms of CST, or the CRI, and the ailments to which it is commonly applied, including those evaluated in this meta-analysis. Additionally, despite regular application, there is limited indication for manipulation of this type in the pediatric population.

Despite significant controversy over its continued use, CST has gained widespread acceptance in the osteopathic community (12, 13). It is used especially heavily in Europe, where over 90% of osteopaths report using CST (14–18). In the United States, it is more difficult to estimate the number osteopathic physicians actively employing CST. A 2021 study based on American Osteopathic Association survey data found that only about 46% of currently practicing osteopathic physicians use any osteopathic manipulative medicine (OMM) in their practice, though no attempt was made to provide a breakdown by technique (19). Regardless of its use in day-to-day practice, CST is included in the curriculum of all US osteopathic medical schools and is a core component of the COMLEX-USA licensing exams (20).

Studies evaluating the clinical use of CST have not led to a definitive answer regarding its effectiveness, despite relatively strong evidence against its continued use. Prior meta-analyses show significant effects of OMM for the treatment of neck and back pain, but these studies make no attempt to isolate CST modalities (21, 22). Based on existing reviews, other indications for CST are not well supported (23–26). Interrater reliability for palpation of the CRI has been shown to be low (27). Several prior meta-analyses and systematic reviews exploring the efficacy of CST as a standalone treatment disagree on whether there is enough evidence to recommend it for the treatment of any condition (26, 28–32). A more recently published systematic review and meta-analysis by Ceballos-Laita et al. concluded that no significant effect was found in any of several outcome categories (33).

Prior systematic reviews have become outdated by the publication of additional literature (28, 31), are limited to a single indication (26, 30), relied on a few small studies (32), or conducted a more limited meta-analysis than is presented here (33). This review provides an updated meta-analysis of CST and encompasses the breadth of indications for CST that have been evaluated in scientific literature to answer the question: Does CST performed by experts on non-healthy adults or children as compared to standard of care, sham treatment, or no treatment in any setting provide statistically significant benefit to any quantitative outcome? Due to the wide range of available data investigating CST, multiple meta-analyses of general outcome categories have been compiled and presented here.

## 2 Methods

This study was reported following the guidelines set out in the Preferred Reporting Items for Systematic Reviews and Meta-analysis (PRISMA) (34). This report was pre-registered with Open Science Framework (OSF) Registries (35) and a preprint is available at Research Square (36).

### 2.1 Data sources and search

A search of Embase, PubMed, and Scopus was conducted to identify all randomized controlled trials examining the efficacy of craniosacral techniques, published from database inception to October 29^th^, 2023. An updated search was run on May 8, 2024, to ensure all relevant records were captured prior to publication. The Scopus search was limited to title, abstract, and keyword. A grey literature search using Google Scholar was also conducted using a shortened search term, due to the limitations of the Google Scholar search interface. Additionally, a backward citation search was conducted. The full search term is available via the OSF registration as well as in Supplementary material (Supplementary Table 1). This term was developed with input from a health sciences librarian.

### 2.2 Eligibility criteria

All randomized controlled trials assessing clinical effectiveness of CST performed by experts on non-healthy adults or children as compared to standard of care, sham treatment, or no treatment in any setting with any measured outcome were included. For the purposes of this study, CST is defined as any manual technique that attempts to influence the cranial sutures, dura mater, sacrum, flow of cerebrospinal fluid, or otherwise subscribes to the theories of biomechanics commonly accepted as cranial manipulative medicine (3). Studies that were not available in English, studies that used animal subjects, studies that did not incorporate craniosacral osteopathic medicine, and studies in which healthy participants were treated with CST without indication were excluded. Additionally, studies that combined craniosacral techniques with other non-osteopathic treatments, and did not separate or distinguish results, were excluded. One study meeting this description was included as the initial trial outcome specified separation but post-hoc analysis was conducted on combined groups due to sample limitations. Studies that did not clearly present trial data were excluded, as were studies that did not present data in such a way as to allow meta-analysis. An attempt was made to contact the authors of any paper lacking data that was deemed otherwise relevant to this report; no additional reports were included following these attempts.

### 2.3 Data extraction

Search data were retrieved manually and deduplicated using Deduklick (37). Deduplication was manually verified using EndNote. Deduplicated results were then upload to Rayyan for screening (38). Screening was conducted by three independent reviewers (A.A., A.S., and R.P.). All conflicts were resolved via joint discussion prior to final inclusion.

### 2.4 Quality assessment

Report quality and risk of bias was assessed using the Cochrane Risk of Bias 2 tool (39). The risk of bias was assessed by three independent reviewers. Two reviewers selected from A.S., R.P., A.P., and D.S. were assigned using a random number generator to each included record. A third reviewer (A.A.) conducted an independent review and resolved disputes prior to the final risk of bias assessment.

We assessed the quality of evidence of each outcome category using the Grading of Recommendations Assessment, Development and Evaluation (GRADE) system using GRADEpro GDT (40, 41). One reviewer (A.A.) assigned a GRADE rating (high, moderate, low, very low) to each outcome. Each rating was then evaluated independently by two additional authors (R.P., D.S.) and finalized by group discussion. The final ratings were agreed upon by all authors. Quality of evidence was assessed based on the following domains: risk of bias, inconsistency of results, indirectness of evidence, imprecision of results, publication bias. As our review included only RCTs, quality of evidence was initially rated as “high quality” and downgraded by one level per “serious limitation” identified and by two levels per “very serious limitation” identified. Criteria for downgrading are as follows: risk of bias was downgraded one level if between 50 and 75% of papers contributing to an outcome were rated as “High Risk of Bias” and was downgraded two levels if ≥75% of papers contributing to an outcome were rated as “High Risk of Bias.” Inconsistency of results was downgraded by one level if the outcome had an I^2^ of between 50 and 75% and was downgraded by two levels if I^2^ was ≥75%. Indirectness of evidence was downgraded based on the presence of confounding factors in the study designs or populations studied, for example, if the studies included in an outcome did not isolate the intervention. Imprecision of results was downgraded by one level if the confidence interval crossed zero. Summary of recommendations tables may be viewed in Supplementary Tables 2, 3.

### 2.5 Outcomes

The primary outcome was any statistically significant change in treatment outcomes after treatment with CST. All treatment outcomes presented by the retrieved reports were considered for this study. The conditions treated by CST in the evaluated studies were not sufficiently homogenous to allow for a single meta-analysis, so clinical outcomes were grouped into categories and multiple meta-analyses were conducted (Table 1). All results pertaining to these listed outcomes were retrieved. Outcomes were excluded if all screening authors (A.A., A.S., R.P., A.P., D.S.) agreed that they measured unrelated physiologic changes with no evidence of relationship to either the condition for which treatment was sought or to the treatment itself. No demographic data were compiled from the included reports.

**Table 1:** Description of outcome categories.

Outcome category	Description	Studied contributed
Disability	Metrics quantifying the impact a pathology has on daily living activities, based on disability scores	Castro-Sánchez et al. (61), Duncan et al. (63), Elden et al. (64), Haller et al. (65), Muñoz-Gómez et al. (71), Rolle et al. (74)
Disease incidence	Refers to the onset or recurrence of a specific disease state	Wahl et al. (79)
Mental function	Measurements of the mental capacity based on one of several tests	Accorsi et al. (57), Castro-Sánchez et al. (61), Duncan et al. (63), Rolle et al. (74), Wyatt et al. (80)
Mental health	Quantifies the psychological state and well-being of an individual	Elden et al. (64), Haller et al. (65), Matarán-Peñarrocha et al. (69), Muñoz-Gómez et al. (71), Wyatt et al. (80)
Motor function	Motor function as assessed by one of several gross motor function scales	Castro-Sánchez et al. (61), Duncan et al. (63), Matarán-Peñarrocha et al. (69), Terrell et al. (77), Wyatt et al. (80)
Movement	Assessment of specific movements	Duncan et al. (63), Wyatt et al. (80)
Neonate health, behavior	Refers to neonate actions, such as feeding, sleeping, and colic	Castejón-Castejón et al. (59), Cerritelli et al. (62), Hayden et al. (67), Herzhaft Le Roy et al. (68), Raith et al. (73), Vandenplas et al. (78)
Neonate health, structure	Defined by somatic aspects of neonate health, namely head shape.	Bagagiolo et al. (58), Philippi et al. (72)
Pain, chronic somatic	Refers to the symptoms caused by non-specific recurrent pain	Castro-Sánchez et al. (60), Castro-Sánchez et al. (61), Duncan et al. (63), Elden et al. (64), Haller et al. (65), Matarán-Peñarrocha et al. (69), Mazreati et al. (70), Wyatt et al. (80)
Pain, headache	Evaluates symptoms induced by non-specific headaches	Hanten et al. (66), Muñoz-Gómez et al. (71), Rolle et al. (74)
Pain, headache medication use	Measurements of the incidence of abortive headache medication usage	Muñoz-Gómez et al. (71), Rolle et al. (74)
Quality of life	Quantifies the degree of general suffering an individual is experiencing	Haller et al. (65), Matarán-Peñarrocha et al. (69), Muñoz-Gómez et al. (71)
Sleep	Related to elements of adult or adolescent sleep health and quality	Matarán-Peñarrocha et al. (69), Wyatt et al. (80)
Vision	Evaluation of visual performance and eye health	Sandhouse et al. (75), Sandhouse et al. (76)

### 2.6 Data synthesis and analysis

Data extraction was completed by a single author (A.A.) and independently verified by a second author (R.P.). Data analysis was completed by a single author (A.A.) and independently verified by a second author (D.S.). Data were manually extracted from all included trials. All pooling and meta-analysis was conducted with R software utilizing the “*metafor*,” “*dmetar*,” “*meta*,” and “*metasens*” packages (42–45). General analysis followed the plan laid out in the pre-registration and consisted of effect size calculation, outlier analysis, heterogeneity analysis, publication bias analysis, and risk of bias assessment. Modification was made to the pre-specified plan as data did not allow for analysis without subdivision. No aggregate effect size is presented due to study heterogeneity and outcome variability. Per study effect size has been reported based on the first time point recorded for the primary outcome only. Subgroup analysis based on outcome category was conducted on primary outcomes at the first measured time point. Additionally, primary and secondary outcomes at all time points were included in a separate per-outcome analysis.

Manual effect size calculation was performed if the RCT did not present effect sizes. To account for different scales within outcome measurement, effect sizes were calculated as mean differences using Hedge’s G values. All outcomes were continuous. Outcomes presented as odds ratio were converted to mean differences. An inverse variance random effect model with HKSJ variation correction was used to combine effect sizes in each outcome category as well as for subgroup analysis of primary outcomes. Outcome heterogeneity was assessed using I^2^ and tau calculated using a Sidik-Jonkman estimator.

Outlier studies were identified by comparing individual aggregate effect size confidence intervals with the pooled effect size confidence interval; non-overlapping confidence intervals were flagged as potential outliers. Outliers were further explored by performing influence analysis and generating a Baujat plot. Per-outcome subgroup analysis was performed including and excluding outliers.

To further explore potential publication bias and p-hacking in significant outcomes, p-curve analysis was conducted following the methodology described by Simonsohn et al. (46). This method attempts to assess publication bias more reliably than a single significant *p*-value by investigating the plotted curve of all significant *p*-values. Positive results may be said to have “evidential value,” indicating a true effect.

All code and data used in this meta-analysis are available on GitHub via Zenodo at https://zenodo.org/doi/10.5281/zenodo.10022853.

## 3 Results

### 3.1 Study selection

**Figure 1:**
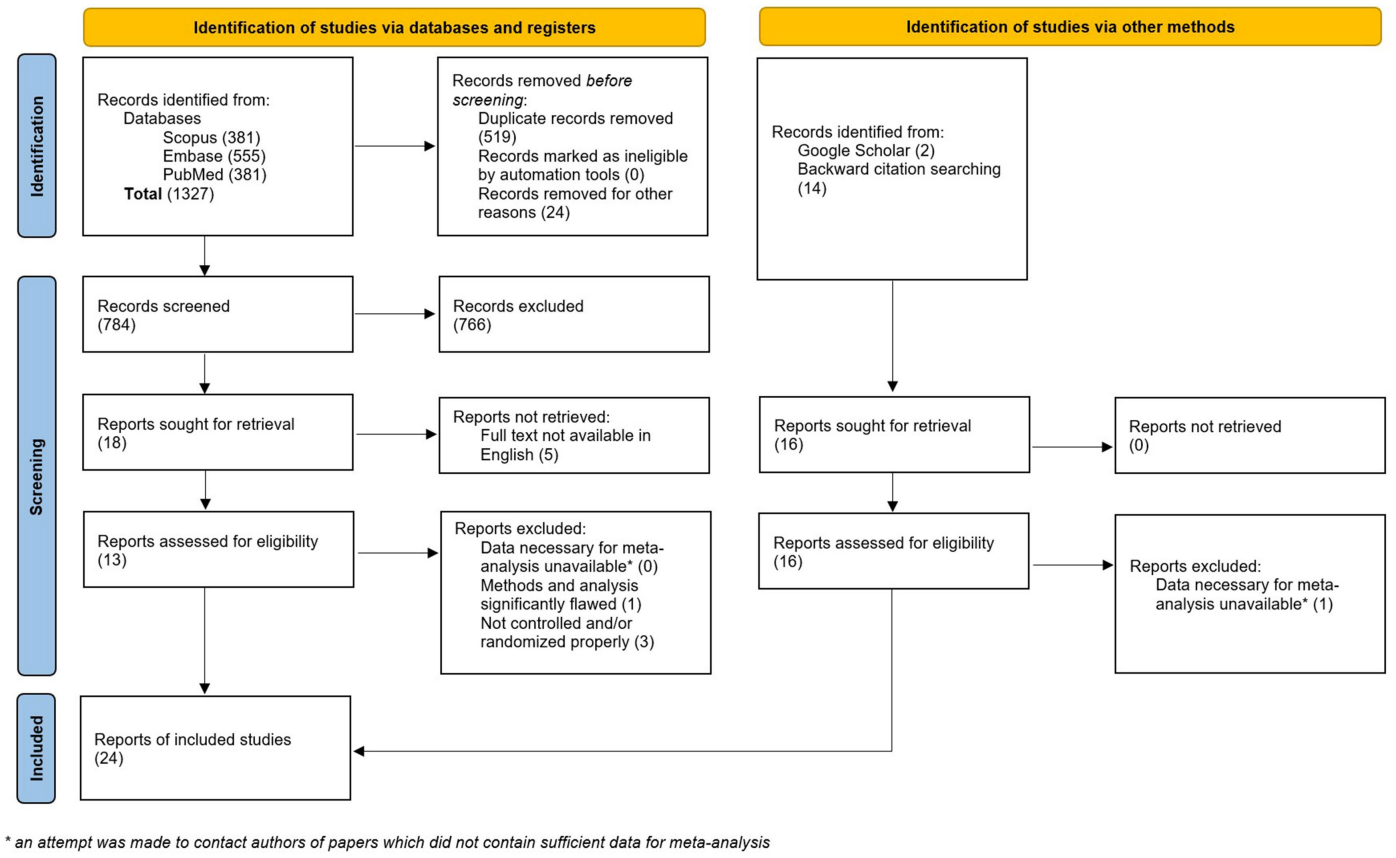
PRISMA flow diagram.

A total of 1,192 studies were retrieved after the initial search. After deduplication, 649 unique studies remained. An additional 16 studies were identified via Google Scholar search and backward citation searching. Of the 665 studies, 34 records were sought for retrieval. Of these, five were unavailable in English (47–51), one did not contain sufficient data for meta-analysis (52), and four were not properly randomized or otherwise contained methodological issues that rendered them unsuitable for inclusion in the meta-analysis (53–56) (Figure 1). Twenty-four studies were included in the final analysis with a total of 1,613 participants (57–80) (Table 2). An updated search on 5/8/2024 retrieved an additional 134 records, none of which met our inclusion criteria.

**Table 2:** Summary of included studies.

Study	Analysis plan (intention to treat vs. per protocol)	Sample size (experimental/control)	Experimental intervention	Control intervention	Outcome	Risk of bias	Results
Accorsi et al. (57)	Per-Protocol	28 (14/14)	Craniosacral Therapy	Conventional Care	ADHD Treatment	High	Multivariate linear regression showed that OMT was positively associated with changes in the Biancardi-Stroppa Test accuracy and rapidity scores.
Bagagiolo et al. (58)	Intention-to-Treat	96 (48/48)	OMT (including Craniosacral Therapy) + Repositioning Therapy	Sham Treatment + Repositioning Therapy	Neonate Cranial Asymmetry Treatment	Low	Multivariate logistical regression showed that OMT was positively associated with a reduction in ODDI scores
Castejón-Castejón et al. (59)	Per-Protocol	54 (29/25)	Craniosacral Therapy	No Treatment	Infantile Colic Treatment	High	ANCOVA with a Bonferroni post-hoc correction showed that craniosacral therapy was positively associated with a reduction in crying hours, an increase in hours of sleep, and a decrease in colic severity measured by the Infantile Colic Severity Questionnaire
Castro-Sánchez et al. (60)	Per-Protocol	92 (46/46)	Craniosacral Therapy	Sham Treatment	Fibromyalgia Treatment	High	Paired two-sample t-tests showed that craniosacral therapy was positively associated with a reduction in pain of tenderpoints, temporal standard deviation of RR segments, root mean square deviation of temporal standard deviation of RR segments, and clinical global impression of improvement
Castro-Sánchez et al. (61)	Intention-to-Treat	64 (32/32)	Craniosacral Therapy	Massage Therapy	Chronic Low Back Pain Treatment	Low	ANCOVA showed no significant difference in the Roland Morris Disability Questionnaire results
Cerritelli et al. (62)	Intention-to-Treat	110 (55/55)	OMT (including Craniosacral Therapy) + Conventional Care	Conventional Care	Length of Stay in Hospitals for Premature Infants	Low	A generalized linear model showed that OMT was positively associated with a length of hospital stay reduction in premature infants
Duncan et al. (63)	Per-Protocol	55 (19/17/19)	Group 1: OMT (including Craniosacral Therapy) Group 2: Acupuncture Treatment	No Treatment	Cerebral Palsy Treatment	High	Hierarchical linear regression models showed a positive association for OMT but not acupuncture treatment in improved Gross Motor Function Measurement score and the mobility domain in the Functional Independence Measure score
Elden et al. (64)	Intention-to-Treat	123 (63/60)	Craniosacral Therapy + Conventional Care	Conventional Care	Pelvic Girdle Pain Treatment and Sick Leave Time in Pregnant Women	High	Mann–Whitney U-tests showed that OMT combined with conventional care had a positive association with a reduction in pelvic girdle pain in the morning but a non-significant impact in a reduction of pelvic girdle pain in the evening or sick leave time
Haller et al. (65)	Intention-to-Treat	54 (27/27)	Craniosacral Therapy	Sham Treatment	Chronic Neck Pain Treatment	Low	Univariate analysis of covariance showed that craniosacral therapy was positively associated with a reduction of neck pain intensity
Hanten et al. (66)	Intention-to-Treat	60 (20/20/20)	Group 1: Resting Position Technique Treatment Group 2: Craniosacral Therapy	No Treatment	Tension-type Headache Treatment	High	One-way MANCOVA followed by univariate and post-hoc tests showed that craniosacral therapy but not resting position technique treatment had a positive association with a reduction in pain intensity during an attack
Hayden et al. (67)	Per-Protocol	28 (14/14)	Craniosacral Therapy	No Treatment	Infantile Colic Treatment	High	Paired two-sample t-tests showed that craniosacral therapy was positively associated with a reduction in hours spent crying and an increase in hours spent sleeping
Herzhaft Le Roy et al. (68)	Intention-to-Treat	97 (49/48)	OMT (including Craniosacral Therapy)	Sham Treatment	Neonate Biomechanical Suckling Ability	Low	Longitudinal regression models showed that OMT was positively associated with an improvement in LATCH scores
Matarán-Peñarrocha et al. (69)	Per-Protocol	84 (43/41)	Craniosacral Therapy	Sham Treatment	Fibromyalgia Treatment	High	Paired two-sample t-tests showed that craniosacral therapy was positively associated with a reduction in pain and an improvement in Pittsburgh Sleep Quality Index, short form-36 health survey, Beck depression inventory, and State Trait Anxiety Inventory scores
Mazreati et al. (70)	Per-Protocol	59 (30/29)	Craniosacral Therapy	Sham Treatment	Chronic Back Pain Treatment in Nurses	High	ANCOVA showed that craniosacral therapy had a positive association with an improvement in McGill Pain Questionnaire scores
Muñoz-Gómez et al. (71)	Intention-to-Treat	50 (25/25)	Craniosacral Therapy	Sham Treatment	Migraine Treatment	Some Concern	Two-factor mixed MANCOVA showed that craniosacral therapy was positively associated with a reduction in pain and pain medication intake as well as an improvement in Headache Disability Index and Patients’ Global Impression of Change scores
Philippi et al. (72)	Intention-to-Treat	32 (16/16)	OMT (including Craniosacral Therapy)	Sham Treatment	Neonate Postural Asymmetry Treatment	Low	Independent t-tests showed that OMT was positively associated with an improvement in standardized asymmetry scores
Raith et al. (73)	Intention-to-Treat	25 (12/13)	Craniosacral Therapy	Conventional Care	Neurological Development in Premature Neonates	Low	First order autoregressive covaraince structure calculations showed no significant difference in global General Movement Assessment scores
Rolle et al. (74)	Per-Protocol	40 (21/19)	OMT (including Craniosacral Therapy)	Sham Treatment	Frequent Episodic Tension-type Headache Treatment	High	2-way ANOVA followed by a multiple comparison Tukey test showed that OMT was positively associated with a reduction in headache frequency
Sandhouse et al. (76)	Per-Protocol	89 (47/42)	Craniosacral Therapy	Sham Treatment	Visual Function	High	Hierarchical ANOVA showed that craniosacral therapy was positively associated with an effect on pupillary size under bright light in the left eye and in near point of convergence break but no significance was found with pupillary size under bright light in the right eye, pupillary size under dim light in both eyes, best-corrected distance visual acuity testing in both eyes, Donder pushup testing in both eyes, near point of convergence recovery, or the cover test with prism neutralization
Sandhouse et al. (75)	Per-Protocol	29 (15/14)	Craniosacral Therapy	Sham Treatment	Visual Function	High	Hierarchical ANOVA showed that craniosacral therapy was positively associated with an effect on pupillary size under bright light in the right eye but no significance was found with pupillary size under bright light in the left eye, pupillary size under dim light in both eyes, best-corrected distance visual acuity testing in both eyes, Donder pushup testing in both eyes, near point in convergence break and recovery, or the cover test with prism neutralization
Terrell et al. (77)	Intention-to-Treat	84 (15/15/13/15/14/12)	Parkinson’s Patients: Group 1: “Whole-body” OMT (including Craniosacral Therapy) Group 2: “Neck-down” OMT Group 3: Sham Treatment	Healthy age-matched controls: Group 1: “Whole-body” OMT (including Craniosacral Therapy) Group 2: “Neck-down” OMT Group 3: Sham Treatment	Parkinsonian Gait Treatment	High	Paired two-sample t-tests and waveform analysis show that craniosacral therapy in conjunction with OMT but not OMT alone or the sham treatment was positively associated with reduced hip extension in the mid-to-late stance phase and reduced knee extension in the stance phase in Parkinsons patients compared to controls but craniosacral therapy in conjunction with OMT, OMT alone, and the sham treatmet had no significance on saggital hip, knee, or ankle angles througout the gait cycle in Parkinsons patients compared to controls
Vandenplas et al. (78)	Per-Protocol	28 (15/13)	OMT (including Craniosacral Therapy)	Sham Treatment	Obstructive Apnea Treatment in Neonates	Some Concern	Mann–Whitney U-tests showed that OMT was positively associated with a decrease in obstructive apneas measured via polysomnographs
Wahl et al. (79)	Intention-to-Treat	90 (24/22/22/22)	Group 1: OMT (including Craniosacral Therapy) with Sham Echinacea Treatment Group 2: Echinacea Treatment with Sham OMT Treatment Group 3: OMT (including Craniosacreal Therapy) with Echinacea Treatment	Sham OMT and Sham Echinacea Treatment	Recurrent Otitis Media Treatment in Young Children	High	Mann- Whitnet U-tests showed no significance with OMT and the reduction of risk of acute otitis media, no significant interaction between OMT and Echinacea treatment, and that Echinacea treatment was negatively associated with a reduction of risk of acute otitis media
Wyatt et al. (80)	Intention-to-Treat	142 (71/71)	Craniosacral Therapy	No Treatment	Cerebral Palsy Treatment	Some Concern	Generalized linear modeling procedures and analysis showed no significance with OMT and change in Gross Motor Function Measure-66 and Child Health Questionnaire PF50 scores

### 3.2 Risk of bias

**Figure 2:**
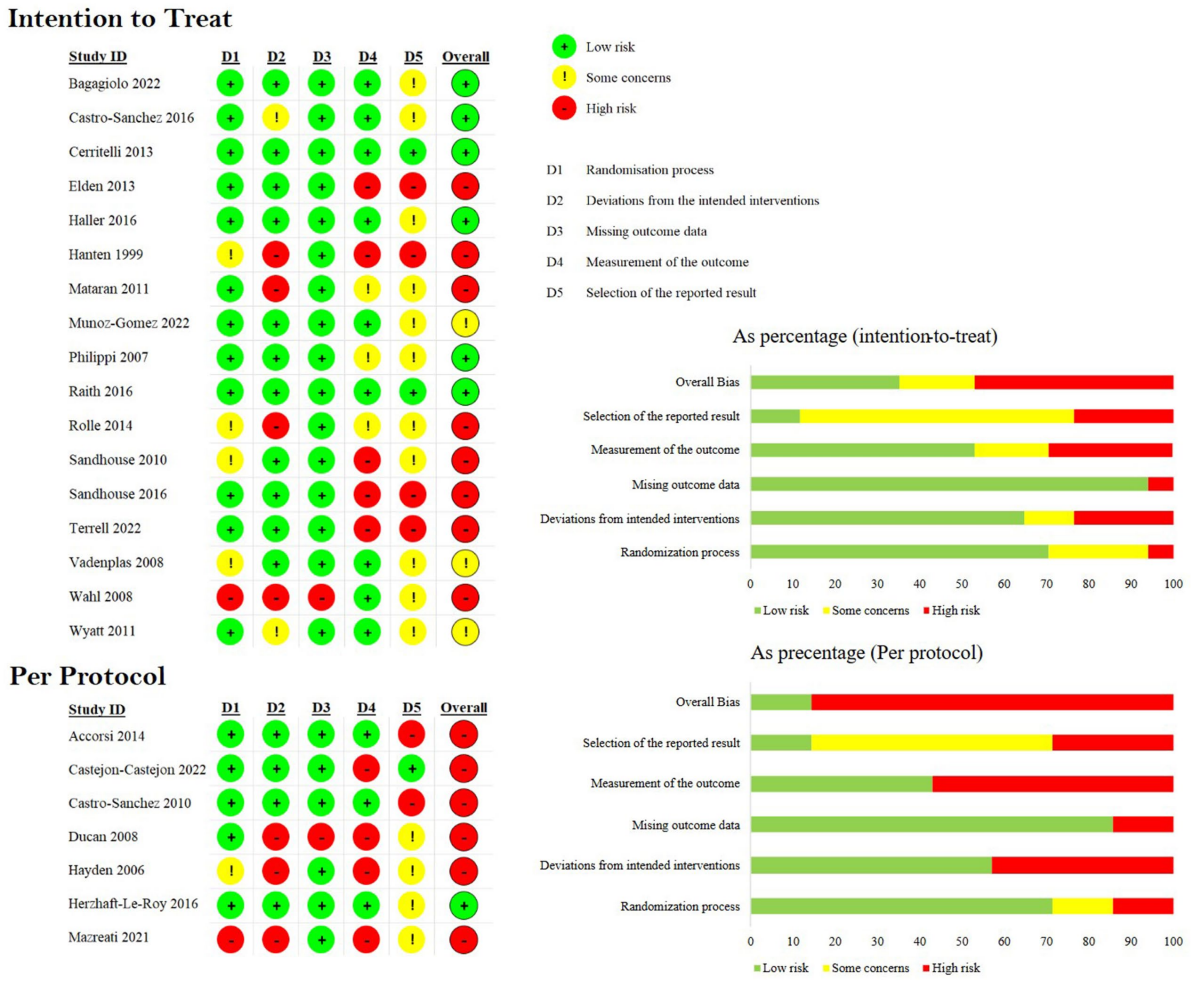
Risk of bias. Studies have been divided based on stated analysis plan. Seven studies were identified as “Low risk,” three as “Some concerns,” and fourteen as “High risk” for bias.

Of the 24 included studies, 14 were rated as “High Risk.” Three additional studies were rated as “Some Concern” (Figure 2). The primary findings that increased bias included following a per-protocol analysis plan, deviations from planned interventions, inconsistent outcome reporting, and statistical analysis that appeared to be post-hoc or otherwise inappropriate given the experimental design. Additionally, small sample sizes in all the studies and lack of power calculations contribute to bias rating. All but two studies were marked either “Some Concern” or “High Risk” in their selection of reported results. Complete and transparent data were provided by 22 of the 24 studies, though this is likely biased by the fact that studies not containing sufficient data were excluded from this meta-analysis.

### 3.3 Outliers

**Figure 3:**
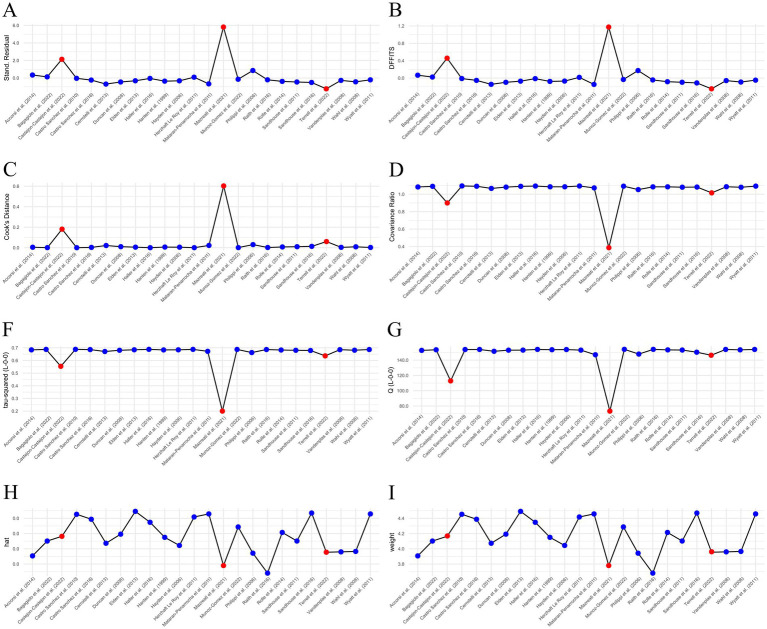
Influence analysis. Both Castejón-Castejón et al. (59) and Mazreati et al. (70) stand out as potential outliers. Terrell et al. (77) is not as convincing. **(A)** External residuals calculated with a leave-one-out method. **(B)** Difference in fits (DFFITS). **(C)** Cook’s distance. **(D)** Covariance ratio. **(F,G)** Leave-one-out Tau and Cochrane’s Q. **(H)** Hat value. **(I)** Study weights. Potential outliers are highlighted in red.

**Figure 4:**
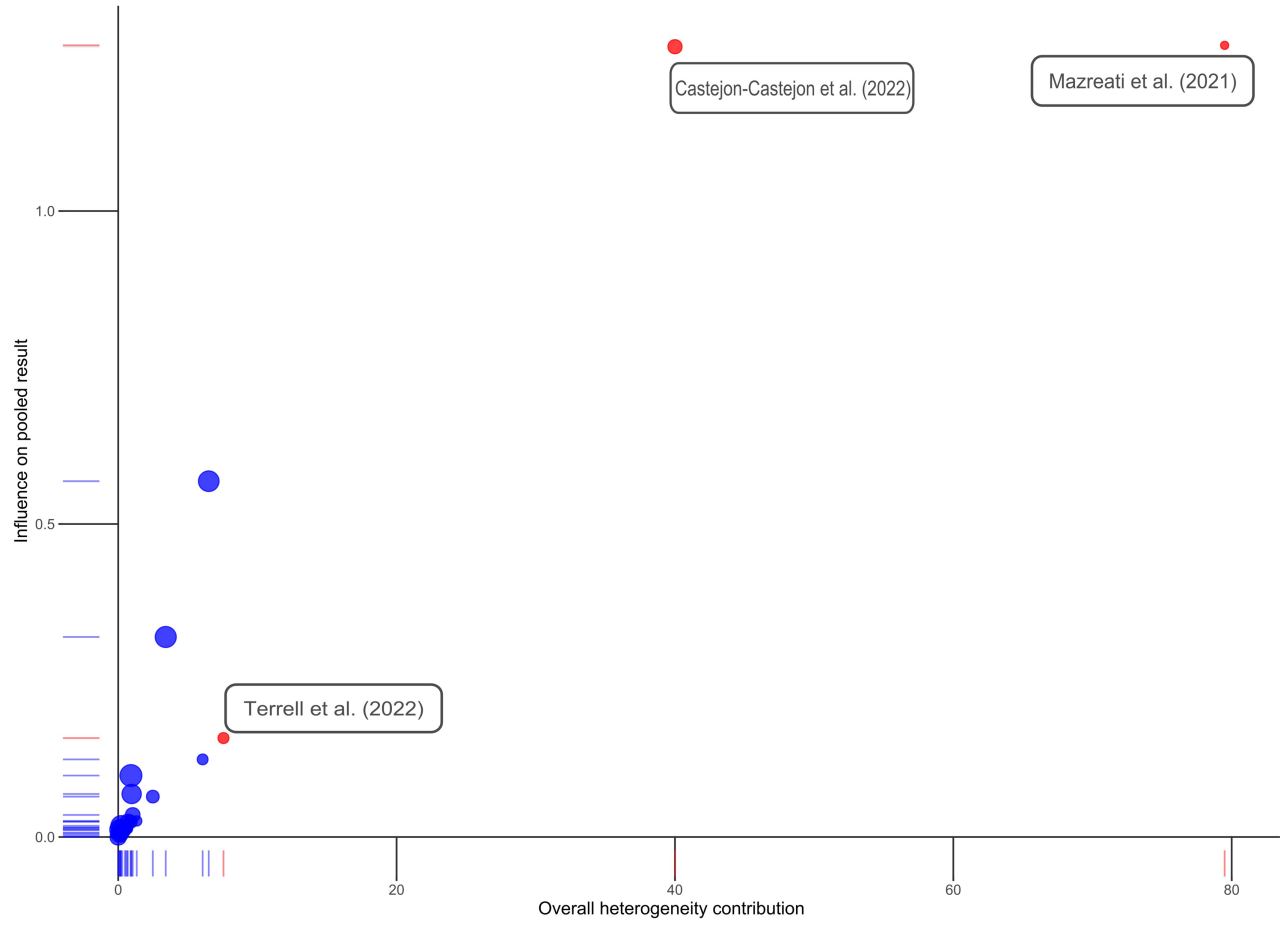
Baujat plot. Studies initially identified as possible outliers are colored in red and labeled. Note that Terrell et al. (77) does not appear to be a true outlier.

Three outliers were identified: Castejón-Castejón et al. (59), Mazreati et al. (70), and Terrell et al. (77). All three studies had been marked “High Risk of Bias.” Castejón-Castejón et al. (59) and Mazreati et al. (70) both displayed unusually high effect sizes in the positive direction. To confirm outliers, an influence analysis was conducted and a Baujat plot generated both of which support removal of identified articles (Figures 3, 4). While initial heterogeneity was presumed to be a result of between-study variation, given the significant reduction in heterogeneity when outliers were removed (with outliers *I^2^* = 91.21%; without outliers *I^2^* = 14.8%), the decision was made to partially conduct analysis excluding the most extreme outliers. This resulted in the exclusion of two articles: Castejón-Castejón et al. (59) and Mazreati et al. (70).

### 3.4 Effect size estimation

**Figure 5:**
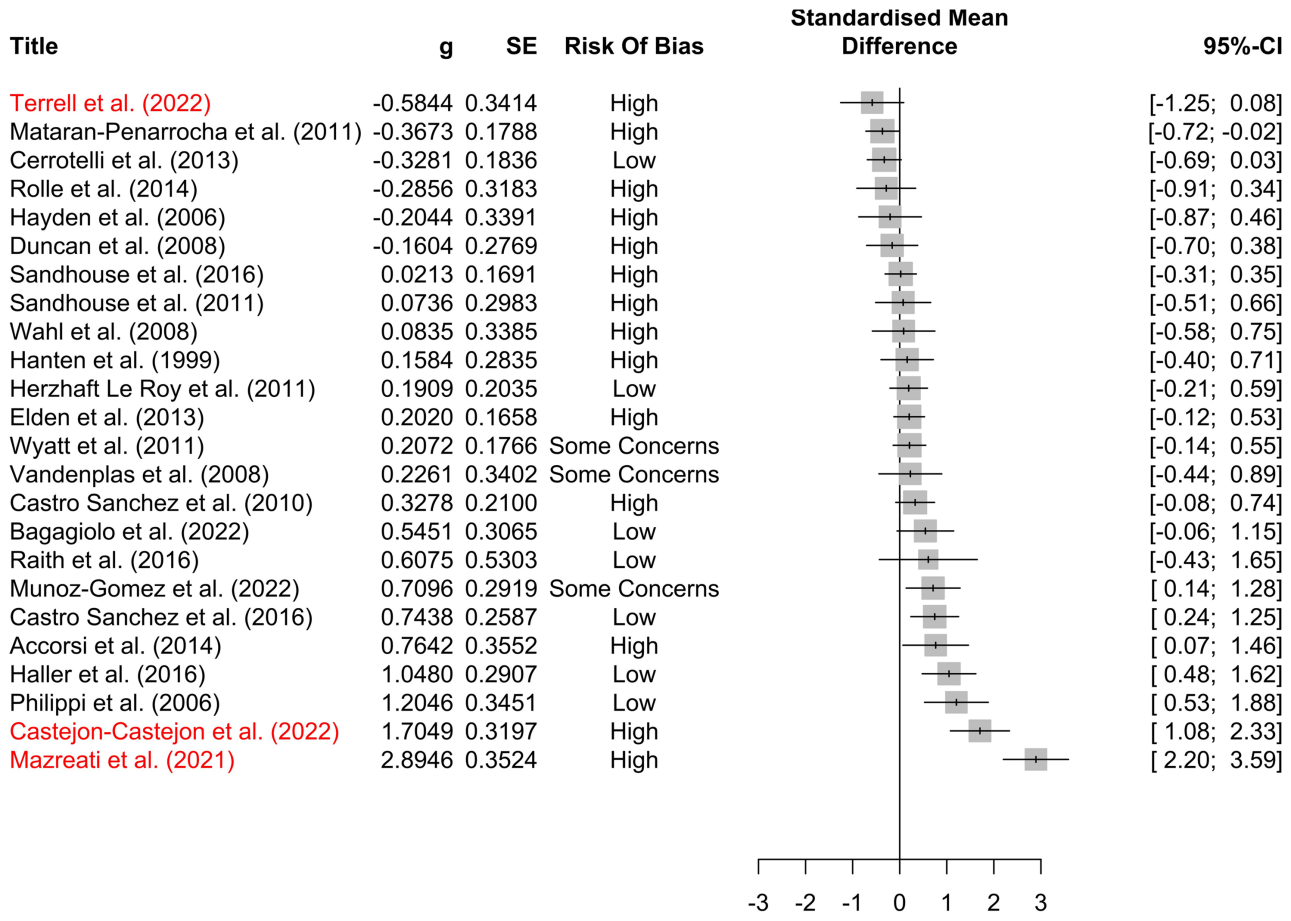
Per-study effect sizes of primary outcomes. Effect sizes were calculated using the first timepoint of the primary outcome. Effect sizes are displayed as *g* including a 95% confidence interval, standard error, and risk of bias. Potential outlier studies have been highlighted in red. Positive effect size favors treatment, negative effect size favors comparison.

**Figure 6:**
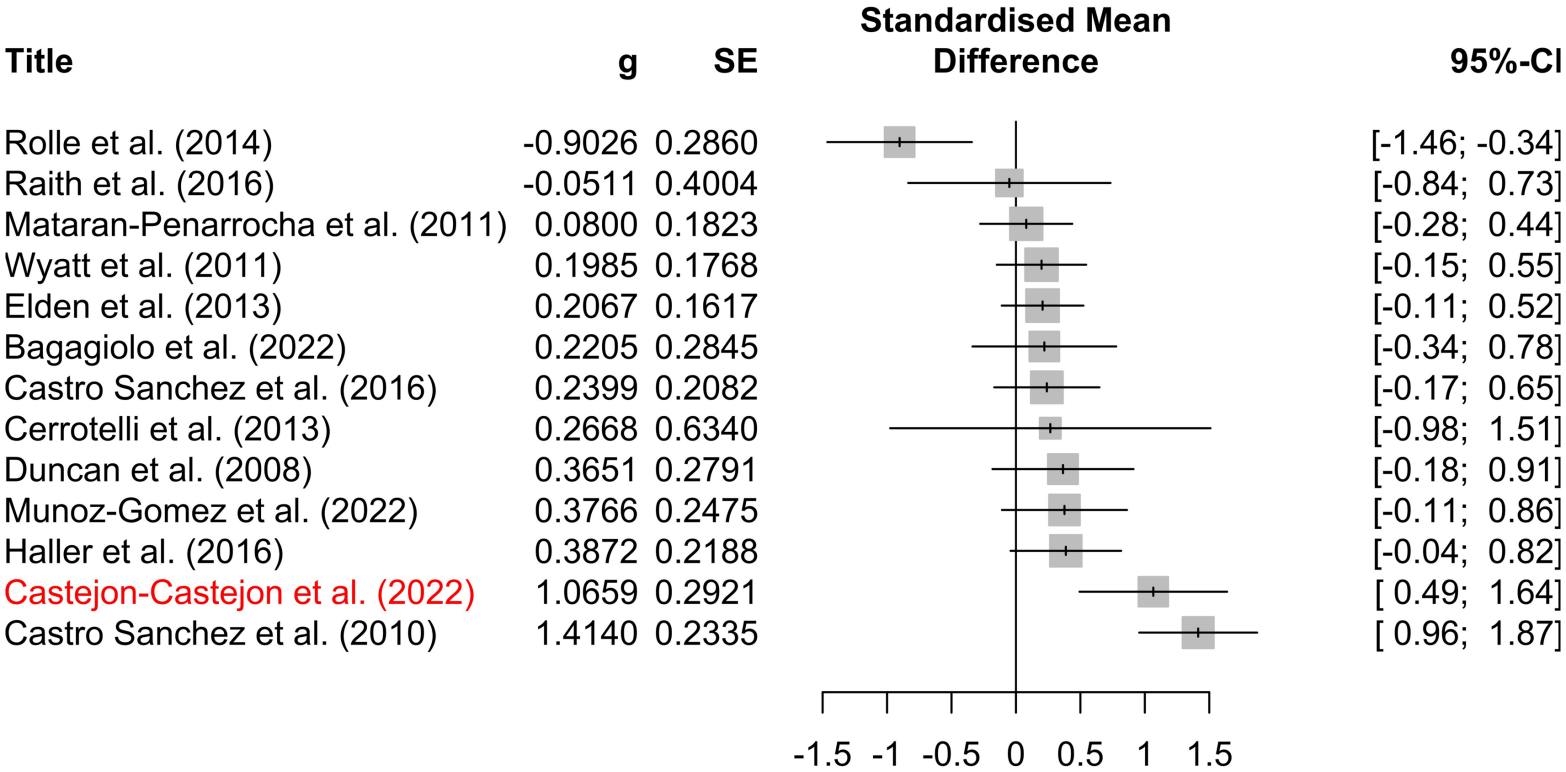
Per-study effect sizes of secondary outcomes. Effect sizes were calculated using the first timepoint of the secondary outcome. Effect sizes are displayed as *g* including a 95% confidence interval, standard error, and risk of bias. Potential outlier studies have been highlighted in red. Positive effect size favors treatment, negative effect size favors comparison.

**Figure 7:**
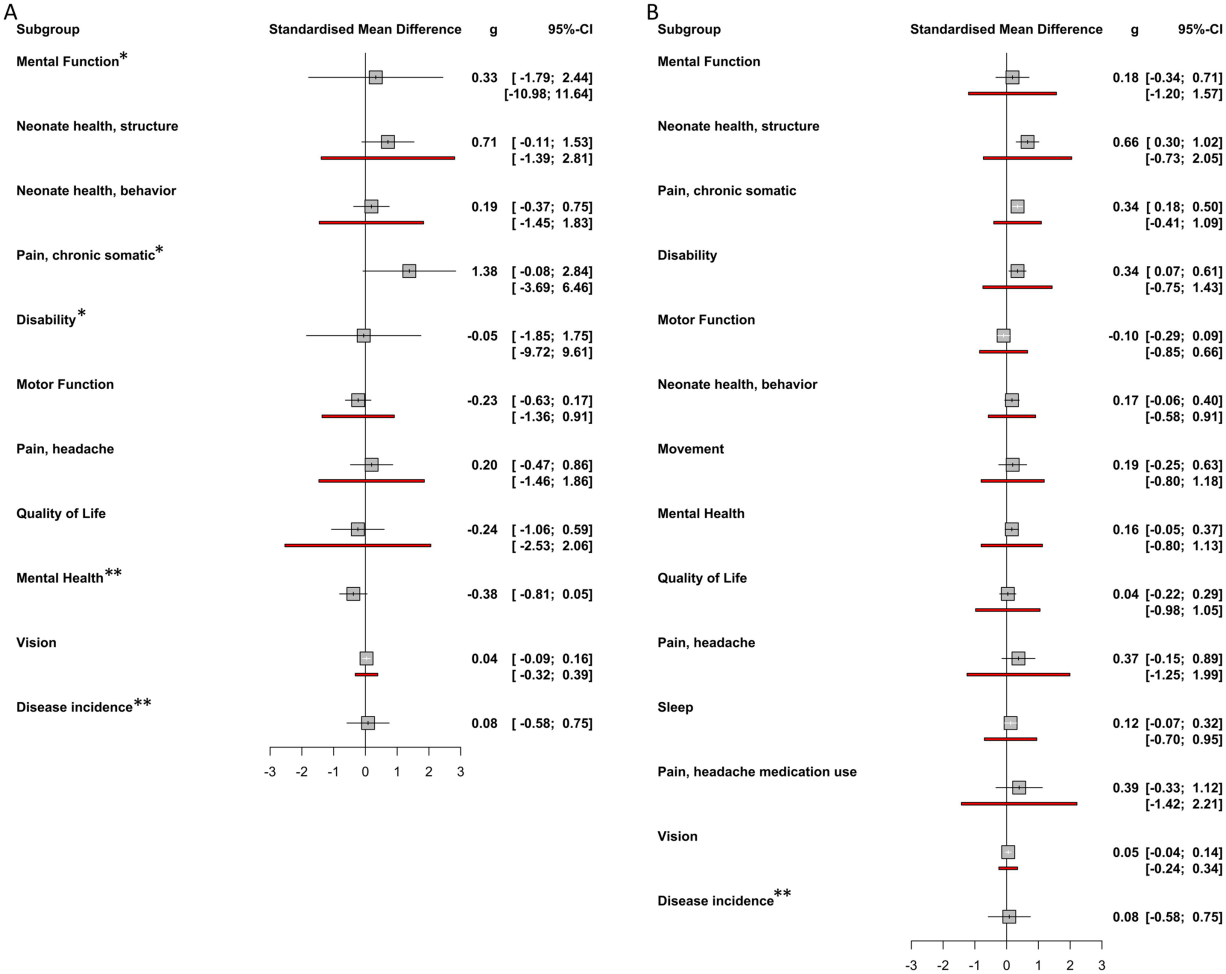
Per-outcome subgroup analysis effect sizes. **(A)** Overall effect sizes for each primary outcome subgroup calculated using random effects models. No significant effects were found for any outcome subgroup. **(B)** Overall effect sizes for each outcome subgroup calculated using random effects models. These subgroups include both primary and secondary outcomes. *Neonate health, structure*, *Pain, chronic somatic*, and *Disability* were found to have significant effect sizes. Positive affect sizes favor treatment, negative effect sizes favor comparison group. Red lines indicate prediction intervals (PI). Note that some outcome groups do not include both primary and secondary outcomes and so do not appear in both forest plots. * Indicates that prediction interval extends past the bounds of the forest plot. ** Indicates that insufficient observations were available to calculate prediction interval.

The included studies yielded 249 individual effect sizes. When effect sizes of primary outcomes were aggregated on a per-study basis, 17 of 24 (71%) of confidence intervals crossed zero, indicating a non-significant effect (Figure 5). Thirteen of the included studies reported discrete secondary outcomes, 11 of which (85%) were non-significant (Figure 6). Subgroup analysis performed on primary outcomes of all included studies indicated that no significant effect existed in any category (Figure 7A). When effect sizes were aggregated by outcome, including secondary outcomes and multiple time points, non-outlier studies support a significant effect in *Disability*; *Neonate health, structure;* and *Pain, chronic somatic* (Figure 7B). Prediction intervals for all significant per-outcome subgroup analyses substantially cross zero. Expanded results of this analysis, including analysis with outliers included, is available in Supplementary 1.

### 3.5 Publication bias

**Figure 8:**
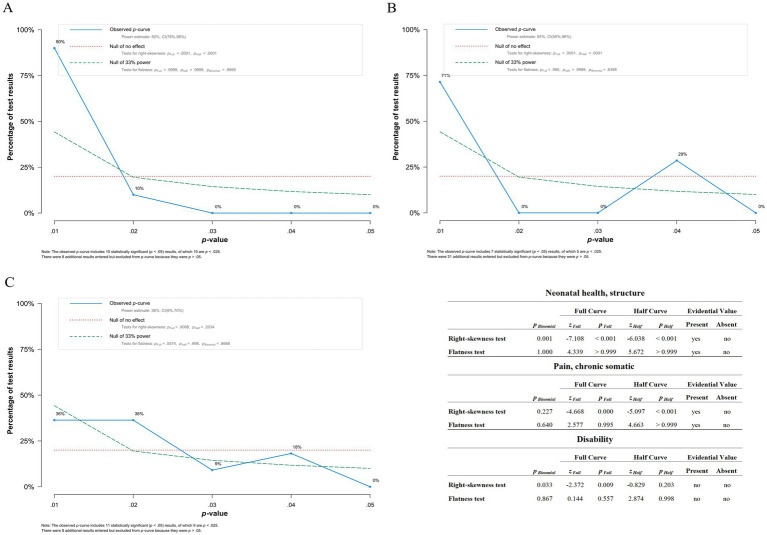
Results of *p* curve analysis. **(A)** *p*-curve of *Neonatal health, structure* showing evidential value. **(B)** *p*-curve of *Pain, chronic somatic* suggesting evidential value. Though the peak at *p* = 0.04 is concerning, more studies data is necessary to determine if it is simply an aberration due to low *n*. **(C)** *p*-curve of Disability suggesting no evidential value, however there is not enough evidence to determine definitively. The included table presents results of flatness and right-skewedness tests used to determine if evidential value is present and/or absent.

P curve analysis conducted on significant outcomes suggests evidential value for *Neonatal health, structure* and *pain, chronic somatic*, implying that the positive findings are not likely due to publication bias. However, *Disability* does not show evidential value (Figure 8).

## 4 Discussion

The existing models describing CST contradict our modern understanding of health and disease, diminishing its plausibility for clinical effectiveness. To establish CST as a recommended practice in clinical settings, compelling results from prospective studies with adequate sample sizes at low risk of bias are necessary. The findings from this meta-analysis indicate that the current body of evidence surrounding CST does not reach this standard. This is in line with conclusions found in previous reviews and meta-analyses (23, 24, 26, 28, 29, 31, 33). A meta-analysis by Haller et al. (30) found a significant effect of CST on pain and disability. However, our subgroup analysis of primary outcomes indicates no significant effects of CST. Only when secondary outcomes are included—arguably a suboptimal approach due to confounding statistical factors—are significant effects identified. Upon close inspection of the meta-analysis by Haller et al., their claims may be based on over-inflated effects likely found due to low quality studies, small sample sizes, under-assessment of bias, and inappropriate grouping of outcomes. Of note, only six of ten studies included in Haller et al. met inclusion criteria and were reanalyzed in this current report. The only other identified positive evidence, presented in a meta-analysis by Jiang et al. (32), found a significant effect of CST on short hamstring syndrome. This indication was not assessed in this report as no related trials meeting inclusion criteria were identified.

While significant findings were reported by most individual studies evaluated here, per-study effect sizes calculated indicate that few significant effects existed. This was true of both individual effect sizes as well as per-study aggregate effect sizes. One possible explanation for this discrepancy is the occurrence of post-hoc statistical analysis and post-hoc outcome selection in the articles—two common concerns identified during the risk of bias assessment. Another contributing factor may be that few studies calculated an effect size or other standardized measure of efficacy. Many of the included studies also did not correct for multiple outcome measures or repeated measures despite high numbers of outcomes and statistical tests.

Notably, our subgroup analysis of primary outcomes shows evidence that there is no significant effect of CST. This result is unsurprising as there is no prior plausibility for a measurable effect on outcomes such as vision, mental health, mental function, quality of life, sleep, movement, or motor function. It is more reasonable that an effect may be observed in subjective measures of pain and disability through the mechanism of the positive effects of touch, which have been shown convincingly in the literature (81). There may also be a plausible mechanism for manipulation of unfused cranial sutures in infants. Even in these populations, however, our evidence suggests no benefit.

Only when all outcomes and timepoints were considered, potential effects on pain, disability, and neonatal structure were observed. Extreme caution should be used when interpreting this as a positive result. While interesting as a statistical exercise, this observation is tempered by various limitations. These limitations include a high level of bias, multiple measures, multiple time points, wide prediction intervals in calculated effect sizes, inadequate blinding and evidence of post-hoc data analysis. Additionally, p-curve analysis of disability-related outcomes does not support true evidential value, though there is not enough evidence to definitively state that none exists. Consequently, caution is advised in interpreting any positive effects identified as the likelihood of significant positive bias affecting the outcomes is considerable.

Despite significant effect sizes and support from p-curve analysis, outcomes related to neonatal structure and somatic pain displayed prediction intervals that broadly crossed zero. Such a wide prediction interval suggests substantial uncertainty regarding the treatment’s potential effectiveness in practical application or in subsequent studies. Thus, these may not represent true positive outcomes and rather reflect statistical aberrations resulting from study limitations. Perhaps even more importantly, these outcome categories are not significant when only primary outcomes are considered. While more research may help clarify whether there is any benefit from the use of CST for these populations and indications, the findings presented here, as well as in prior reviews, suggest a total absence of strong evidence supporting the efficacy of CST.

### 4.1 Limitations

Several important limitations to this analysis exist. The results of the outcome-based subgroup meta-analysis were affected by the decision to exclude outliers. Initially, an algorithm flagged studies in which the effect size fell outside the confidence interval of the group effect size. Influence analysis was conducted in order to confirm these results, which agreed with the initial detection. This process is limited by the variation within and between studies, differing outcomes, and multiple indications. The Supplementary material contains results that include outliers, demonstrating only one significant difference in *Neonate health, behavior* (Supplementary 2C). However, considering the large contribution to heterogeneity and the high bias of the outlier studies, the calculated effect sizes excluding outliers are likely closer to reality.

This analysis is also limited by problems inherent to the included studies. From a statistical perspective, many papers reporting a positive effect failed to account for repeated measures and multiple time point measures. Our own efforts to correct for this based on recommendations by Morris et al. (82) still led to a violation of the assumption of independence, meaning confounding factors may be present. Additionally, a number of the included studies did not provide detailed descriptions of the techniques used and many studies mixed CST with other osteopathic treatments. Rather, precise treatment plans were left to the discretion of the practitioner providing care. Both these factors may have impacted the calculated effect sizes, though likely in the positive direction. What’s more, many included papers suffered from poor blinding, poor randomization, and incomplete result reporting. Given the overall negative outcome, this did not result in reinterpretation of the findings, though care should be taken when interpreting the three positive effects identified.

The limitations in the available literature warrant reservation in considering CST as part of evidence-based treatment plans until substantially higher-quality evidence emerges. Thus, the role and scope of CST should be reevaluated in modern osteopathic medicine. While the findings from this meta-analysis have the potential to inform future research directions, it becomes challenging to advocate for continued exploration of CST considering the predominantly negative outcomes which persist despite various methodological concerns producing probable positive bias. Further research exploring the physiologic mechanisms behind CST could help resolve some of the controversy surrounding its use, though it is unlikely that a plausible biological mechanism will be identified and thus efforts should be directed towards other more promising treatment modalities.

## 5 Conclusion

Craniosacral therapy did not demonstrate broad significance in this meta-analysis, suggesting limited or no usefulness in patient care for a wide range of indications including attention deficit hyperactivity disorder, cranial asymmetry, infant colic, fibromyalgia, low back pain, cerebral palsy, pelvic girdle pain, neck pain, tension-type headache, infant suckling, vision, obstructive sleep apnea, recurrent otitis media, migraine headache, parkinsonian gait, length of stay of premature infants, or neurodevelopment of premature infants. Subgroup analysis of primary outcomes indicates no significant effect when these indications are treated with CST. When secondary outcomes are included, analysis supports possible clinical utility in reducing disability scores, neonatal structural disease, and chronic somatic pain, though methodological limitations substantially reduce the strength of this result. Taken in whole, the currently available literature shows that CST is not effective as a treatment for any investigated condition in either adults or infants. Given the overwhelming evidence that there is no benefit from CST, we do not recommend further exploration of this topic.

## Data Availability

The datasets presented in this study can be found in online repositories. The names of the repository/repositories and accession number(s) can be found here: Github via Zenodo—https://doi.org/10.5281/zenodo.10022854.
