# Supplementary material for: Effectiveness of osteopathic craniosacral techniques: a meta-analysis
Source: Front Med (Lausanne). 2024 Oct 3;11:1452465. doi: 10.3389/fmed.2024.1452465 (PMC11487524; doi:10.3389/fmed.2024.1452465)
Supplement: Supplementary file 1 [file Data_Sheet_1.PDF]

```
##############################################################################################################
Supplement 2A - Primary Outcome Subgroup Analysis
##############################################################################################################

Review: Primary Outcomes

Number of studies: k = 75

Quantifying heterogeneity:
 tau^2 = 0.8808 [0.5091; 1.2753]; tau = 0.9385 [0.7135; 1.1293]
 I^2 = 81.9% [77.8%; 85.2%]; H = 2.35 [2.12; 2.60]

Test of heterogeneity:
      Q d.f.  p-value
 409.00   74 < 0.0001

Results for subgroups (random effects model (HK-CI)):
                                      k       g             95%-CI  tau^2    tau
Q   I^2
Outcome = Mental Function             3  0.3272 [-1.7861; 2.4404] 0.5586 0.7474
10.17 80.3%
Outcome = Neonate health, structure   6  0.7101 [-0.1096; 1.5298] 0.4714 0.6866
34.44 85.5%
Outcome = Neonate health, behavior    9  0.1899 [-0.3690; 0.7487] 0.4226 0.6501
52.69 84.8%
Outcome = Pain, chronic somatic      11  1.3827 [-0.0764; 2.8418] 4.6020 2.1452
168.35 94.1%
Outcome = Disability                  3 -0.0529 [-1.8530; 1.7471] 0.4096 0.6400
12.28 83.7%
Outcome = Motor Function              9 -0.2272 [-0.6289; 0.1745] 0.1982 0.4452
24.76 67.7%
Outcome = Pain, headache              4  0.1962 [-0.4697; 0.8622] 0.0997 0.3158
5.57 46.1%
Outcome = Quality of Life             4 -0.2366 [-1.0647; 0.5914] 0.2180 0.4670
16.43 81.7%
Outcome = Mental Health               1 -0.3788 [-0.8106; 0.0530]     --     --
0.00    --
Outcome = Vision                     24  0.0362 [-0.0894; 0.1618] 0.0250 0.1582
13.52  0.0%
Outcome = Disease incidence           1  0.0835 [-0.5799; 0.7469]     --     --
0.00    --

Test for subgroup differences (random effects model (HK-CI)):
                   Q d.f. p-value
Between groups 16.99   10  0.0747

Details on meta-analytical method:
- Inverse variance method
```

```
- Sidik-Jonkman estimator for tau^2
- Q-Profile method for confidence interval of tau^2 and tau
- Hartung-Knapp (HK) adjustment for random effects model (df = NA)
  (with ad hoc correction)

#####################################################################################
############################
Supplement 2B - Expanded Per-Outcome Meta-analysis Excluding Outliers
#####################################################################################
############################

Review:     Mental Function

Number of studies combined: k = 7

                                  g             95%-CI    t p-value
Random effects model (HK-CI) 0.1846 [-0.3361; 0.7053] 0.87  0.4189

Quantifying heterogeneity:
 tau^2 = 0.2413 [0.0250; 1.5431]; tau = 0.4912 [0.1582; 1.2422]
 I^2 = 67.0% [26.4%; 85.2%]; H = 1.74 [1.17; 2.60]

Test of heterogeneity:
     Q d.f. p-value
 18.16    6  0.0058

Details on meta-analytical method:
- Inverse variance method
- Sidik-Jonkman estimator for tau^2
- Q-Profile method for confidence interval of tau^2 and tau
- Hartung-Knapp (HK) adjustment for random effects model (df = 6)
  (with ad hoc correction)

###############################################################################

Review:     Disability

Number of studies combined: k = 19

                                  g            95%-CI    t p-value
Random effects model (HK-CI) 0.3430 [0.0749; 0.6112] 2.69  0.0150

Quantifying heterogeneity:
 tau^2 = 0.2492 [0.0853; 0.6216]; tau = 0.4992 [0.2921; 0.7884]
 I^2 = 69.1% [50.5%; 80.7%]; H = 1.80 [1.42; 2.28]

Test of heterogeneity:
     Q d.f.  p-value
 58.27   18 < 0.0001
```

```
Details on meta-analytical method:
- Inverse variance method
- Sidik-Jonkman estimator for tau^2
- Q-Profile method for confidence interval of tau^2 and tau
- Hartung-Knapp (HK) adjustment for random effects model (df = 18)
  (with ad hoc correction)

#####################################################################################

Review:     Mental health


         g            95%-CI    z p-value
202 0.0835 [-0.5799; 0.7469] 0.25  0.8052

Details:
- Inverse variance method
Review:     Cranial OMM

Number of studies combined: k = 24

                                  g            95%-CI    t p-value
Random effects model (HK-CI) 0.1633 [-0.0475; 0.3741] 1.60  0.1228

Quantifying heterogeneity:
 tau^2 = 0.2045 [0.0755; 0.4505]; tau = 0.4523 [0.2747; 0.6712]
 I^2 = 69.2% [53.2%; 79.8%]; H = 1.80 [1.46; 2.22]

Test of heterogeneity:
     Q d.f.  p-value
 74.75   23 < 0.0001

Details on meta-analytical method:
- Inverse variance method
- Sidik-Jonkman estimator for tau^2
- Q-Profile method for confidence interval of tau^2 and tau
- Hartung-Knapp (HK) adjustment for random effects model (df = 23)
  (with ad hoc correction)

#####################################################################################

Review:     Motor Function

Number of studies combined: k = 20

                                   g            95%-CI     t p-value
Random effects model (HK-CI) -0.0956 [-0.2857; 0.0945] -0.99  0.3243

Quantifying heterogeneity:
 tau^2 = 0.1201 [0.0239; 0.2969]; tau = 0.3465 [0.1546; 0.5448]
```

```
 I^2 = 58.0% [31.0%; 74.4%]; H = 1.54 [1.20; 1.98]

Test of heterogeneity:
     Q d.f. p-value
 45.21   19  0.0006

Details on meta-analytical method:
- Inverse variance method
- Sidik-Jonkman estimator for tau^2
- Q-Profile method for confidence interval of tau^2 and tau
- Hartung-Knapp (HK) adjustment for random effects model (df = NA)
  (with ad hoc correction)

##############################################################################

Review:     Movement

Number of studies combined: k = 5

                                  g             95%-CI    t p-value
Random effects model (HK-CI) 0.1908 [-0.2474; 0.6289] 1.21  0.2933

Quantifying heterogeneity:
 tau^2 = 0.0654 [0.0000; 0.9603]; tau = 0.2558 [0.0000; 0.9800]
 I^2 = 27.1% [0.0%; 71.2%]; H = 1.17 [1.00; 1.86]

Test of heterogeneity:
    Q d.f. p-value
 5.49    4  0.2409

Details on meta-analytical method:
- Inverse variance method
- Sidik-Jonkman estimator for tau^2
- Q-Profile method for confidence interval of tau^2 and tau
- Hartung-Knapp (HK) adjustment for random effects model (df = 4)
  (with ad hoc correction)

##############################################################################

Review:     Neonatal Health, behavior

Number of studies combined: k = 17

                                  g             95%-CI    t p-value
Random effects model (HK-CI) 0.1679 [-0.0597; 0.3955] 1.56  0.1373

Quantifying heterogeneity:
 tau^2 = 0.1088 [0.0257; 0.2962]; tau = 0.3299 [0.1605; 0.5442]
 I^2 = 63.2% [38.0%; 78.2%]; H = 1.65 [1.27; 2.14]
```

```
Test of heterogeneity:
     Q d.f. p-value
 43.48   16  0.0002

Details on meta-analytical method:
- Inverse variance method
- Sidik-Jonkman estimator for tau^2
- Q-Profile method for confidence interval of tau^2 and tau
- Hartung-Knapp (HK) adjustment for random effects model (df = 16)
  (with ad hoc correction)

##############################################################################

Review:     Neonatal Health, structure

Number of studies combined: k = 18

                                  g           95%-CI    t p-value
Random effects model (HK-CI) 0.6592 [0.3022; 1.0162] 3.90  0.0012

Quantifying heterogeneity:
 tau^2 = 0.3997 [0.1544; 1.0169]; tau = 0.6322 [0.3930; 1.0084]
 I^2 = 77.5% [64.8%; 85.6%]; H = 2.11 [1.69; 2.64]

Test of heterogeneity:
     Q d.f.  p-value
 75.54   17 < 0.0001

Details on meta-analytical method:
- Inverse variance method
- Sidik-Jonkman estimator for tau^2
- Q-Profile method for confidence interval of tau^2 and tau
- Hartung-Knapp (HK) adjustment for random effects model (df = 17)
  (with ad hoc correction)

##############################################################################

Review:     Pain, chronic somatic

Number of studies combined: k = 28

                                  g           95%-CI    t p-value
Random effects model (HK-CI) 0.3439 [0.1829; 0.5049] 4.38  0.0002

Quantifying heterogeneity:
 tau^2 = 0.1262 [0.0483; 0.2583]; tau = 0.3553 [0.2197; 0.5082]
 I^2 = 66.2% [49.8%; 77.3%]; H = 1.72 [1.41; 2.10]

Test of heterogeneity:
     Q d.f.  p-value
```

```
 79.92   27 < 0.0001

Details on meta-analytical method:
- Inverse variance method
- Sidik-Jonkman estimator for tau^2
- Q-Profile method for confidence interval of tau^2 and tau
- Hartung-Knapp (HK) adjustment for random effects model (df = 27)
  (with ad hoc correction)

#############################################################################

Review:     Pain, medication use

Number of studies combined: k = 5

                                  g             95%-CI    t p-value
Random effects model (HK-CI) 0.3947 [-0.3345; 1.1239] 1.50  0.2073

Quantifying heterogeneity:
 tau^2 = 0.2561 [0.0250; 2.7751]; tau = 0.5060 [0.1580; 1.6659]
 I^2 = 71.3% [27.3%; 88.7%]; H = 1.87 [1.17; 2.97]

Test of heterogeneity:
     Q d.f. p-value
 13.93    4  0.0075

Details on meta-analytical method:
- Inverse variance method
- Sidik-Jonkman estimator for tau^2
- Q-Profile method for confidence interval of tau^2 and tau
- Hartung-Knapp (HK) adjustment for random effects model (df = 4)
  (with ad hoc correction)

#############################################################################

Review:     Quality of Life

Number of studies combined: k = 18

                                  g             95%-CI    t p-value
Random effects model (HK-CI) 0.0376 [-0.2170; 0.2921] 0.31  0.7593

Quantifying heterogeneity:
 tau^2 = 0.2136 [0.0834; 0.5414]; tau = 0.4621 [0.2888; 0.7358]
 I^2 = 75.6% [61.6%; 84.6%]; H = 2.03 [1.61; 2.54]

Test of heterogeneity:
     Q d.f.  p-value
 69.78   17 < 0.0001
```

```
Details on meta-analytical method:
- Inverse variance method
- Sidik-Jonkman estimator for tau^2
- Q-Profile method for confidence interval of tau^2 and tau
- Hartung-Knapp (HK) adjustment for random effects model (df = 17)
  (with ad hoc correction)

##############################################################################

Review:     Sleep

Number of studies combined: k = 22

                                  g            95%-CI    t p-value
Random effects model (HK-CI) 0.1249 [-0.0673; 0.3171] 1.35  0.1910

Quantifying heterogeneity:
 tau^2 = 0.1472 [0.0602; 0.3351]; tau = 0.3837 [0.2454; 0.5789]
 I^2 = 72.7% [58.2%; 82.1%]; H = 1.91 [1.55; 2.36]

Test of heterogeneity:
     Q d.f.  p-value
 76.79   21 < 0.0001

Details on meta-analytical method:
- Inverse variance method
- Sidik-Jonkman estimator for tau^2
- Q-Profile method for confidence interval of tau^2 and tau
- Hartung-Knapp (HK) adjustment for random effects model (df = 21)
  (with ad hoc correction)

##############################################################################

Review:     Vision

Number of studies combined: k = 36

                                  g            95%-CI    t p-value
Random effects model (HK-CI) 0.0501 [-0.0422; 0.1424] 1.06  0.2876

Quantifying heterogeneity:
 tau^2 = 0.0187; tau = 0.1368; I^2 = 0.0% [0.0%; 37.8%]; H = 1.00 [1.00; 1.27]

Test of heterogeneity:
     Q d.f. p-value
 20.18   35  0.9787

Details on meta-analytical method:
- Inverse variance method
- Sidik-Jonkman estimator for tau^2
```

```
- Hartung-Knapp (HK) adjustment for random effects model (df = NA)
  (with ad hoc correction)

####################################################################################
############################
Supplement 2C - Expanded Per-Outcome Meta-analysis including Outliers
####################################################################################
############################

Review:     Mental Function

Number of studies combined: k = 7

                                  g             95%-CI    t p-value
Random effects model (HK-CI) 0.1846 [-0.3361; 0.7053] 0.87  0.4189

Quantifying heterogeneity:
 tau^2 = 0.2413 [0.0250; 1.5431]; tau = 0.4912 [0.1582; 1.2422]
 I^2 = 67.0% [26.4%; 85.2%]; H = 1.74 [1.17; 2.60]

Test of heterogeneity:
     Q d.f. p-value
 18.16    6  0.0058

Details on meta-analytical method:
- Inverse variance method
- Sidik-Jonkman estimator for tau^2
- Q-Profile method for confidence interval of tau^2 and tau
- Hartung-Knapp (HK) adjustment for random effects model (df = 6)
  (with ad hoc correction)

##############################################################################

Review:     Disability

Number of studies combined: k = 19

                                  g            95%-CI    t p-value
Random effects model (HK-CI) 0.3430 [0.0749; 0.6112] 2.69  0.0150

Quantifying heterogeneity:
 tau^2 = 0.2492 [0.0853; 0.6216]; tau = 0.4992 [0.2921; 0.7884]
 I^2 = 69.1% [50.5%; 80.7%]; H = 1.80 [1.42; 2.28]

Test of heterogeneity:
     Q d.f.  p-value
 58.27   18 < 0.0001

Details on meta-analytical method:
- Inverse variance method
```

```
- Sidik-Jonkman estimator for tau^2
- Q-Profile method for confidence interval of tau^2 and tau
- Hartung-Knapp (HK) adjustment for random effects model (df = 18)
  (with ad hoc correction)

##############################################################################

Review:     Disease Incidence


         g            95%-CI    z p-value
221 0.0835 [-0.5799; 0.7469] 0.25  0.8052

Details:
- Inverse variance method

##############################################################################

Review:     Mental Health

Number of studies combined: k = 24

                                  g            95%-CI    t p-value
Random effects model (HK-CI) 0.1633 [-0.0475; 0.3741] 1.60  0.1228

Quantifying heterogeneity:
 tau^2 = 0.2045 [0.0755; 0.4505]; tau = 0.4523 [0.2747; 0.6712]
 I^2 = 69.2% [53.2%; 79.8%]; H = 1.80 [1.46; 2.22]

Test of heterogeneity:
     Q d.f.  p-value
 74.75   23 < 0.0001

Details on meta-analytical method:
- Inverse variance method
- Sidik-Jonkman estimator for tau^2
- Q-Profile method for confidence interval of tau^2 and tau
- Hartung-Knapp (HK) adjustment for random effects model (df = 23)
  (with ad hoc correction)

##############################################################################

Review:     Motor Function

Number of studies combined: k = 20

                                   g            95%-CI     t p-value
Random effects model (HK-CI) -0.0956 [-0.2857; 0.0945] -0.99  0.3243

Quantifying heterogeneity:
```

```
 tau^2 = 0.1201 [0.0239; 0.2969]; tau = 0.3465 [0.1546; 0.5448]
 I^2 = 58.0% [31.0%; 74.4%]; H = 1.54 [1.20; 1.98]

Test of heterogeneity:
     Q d.f. p-value
 45.21   19  0.0006

Details on meta-analytical method:
- Inverse variance method
- Sidik-Jonkman estimator for tau^2
- Q-Profile method for confidence interval of tau^2 and tau
- Hartung-Knapp (HK) adjustment for random effects model (df = NA)
  (with ad hoc correction)

##############################################################################

Review:     Movement

Number of studies combined: k = 5

                                  g             95%-CI    t p-value
Random effects model (HK-CI) 0.1908 [-0.2474; 0.6289] 1.21  0.2933

Quantifying heterogeneity:
 tau^2 = 0.0654 [0.0000; 0.9603]; tau = 0.2558 [0.0000; 0.9800]
 I^2 = 27.1% [0.0%; 71.2%]; H = 1.17 [1.00; 1.86]

Test of heterogeneity:
    Q d.f. p-value
 5.49    4  0.2409

Details on meta-analytical method:
- Inverse variance method
- Sidik-Jonkman estimator for tau^2
- Q-Profile method for confidence interval of tau^2 and tau
- Hartung-Knapp (HK) adjustment for random effects model (df = 4)
  (with ad hoc correction)

##############################################################################

Review:     Neonate health, behavior

Number of studies combined: k = 26

                                  g            95%-CI    t p-value
Random effects model (HK-CI) 0.8473 [0.4082; 1.2863] 3.97  0.0005

Quantifying heterogeneity:
 tau^2 = 1.0672 [0.5994; 2.1377]; tau = 1.0330 [0.7742; 1.4621]
 I^2 = 90.4% [87.2%; 92.8%]; H = 3.23 [2.80; 3.74]
```

```
Test of heterogeneity:
      Q d.f.  p-value
 261.04   25 < 0.0001

Details on meta-analytical method:
- Inverse variance method
- Sidik-Jonkman estimator for tau^2
- Q-Profile method for confidence interval of tau^2 and tau
- Hartung-Knapp (HK) adjustment for random effects model (df = 25)
  (with ad hoc correction)

##############################################################################

Review:     Neonate health, structure

Number of studies combined: k = 18

                                  g           95%-CI    t p-value
Random effects model (HK-CI) 0.6592 [0.3022; 1.0162] 3.90  0.0012

Quantifying heterogeneity:
 tau^2 = 0.3997 [0.1544; 1.0169]; tau = 0.6322 [0.3930; 1.0084]
 I^2 = 77.5% [64.8%; 85.6%]; H = 2.11 [1.69; 2.64]

Test of heterogeneity:
     Q d.f.  p-value
 75.54   17 < 0.0001

Details on meta-analytical method:
- Inverse variance method
- Sidik-Jonkman estimator for tau^2
- Q-Profile method for confidence interval of tau^2 and tau
- Hartung-Knapp (HK) adjustment for random effects model (df = 17)
  (with ad hoc correction)

##############################################################################

Review:     Pain, chronic somatic

Number of studies combined: k = 38

                                  g           95%-CI    t p-value
Random effects model (HK-CI) 1.2117 [0.5902; 1.8333] 3.95  0.0003

Quantifying heterogeneity:
 tau^2 = 3.4902 [2.1731; 6.0590]; tau = 1.8682 [1.4742; 2.4615]
 I^2 = 92.9% [91.2%; 94.3%]; H = 3.76 [3.37; 4.20]

Test of heterogeneity:
```

```
      Q d.f.  p-value
 523.83   37 < 0.0001

Details on meta-analytical method:
- Inverse variance method
- Sidik-Jonkman estimator for tau^2
- Q-Profile method for confidence interval of tau^2 and tau
- Hartung-Knapp (HK) adjustment for random effects model (df = 37)
  (with ad hoc correction)

#############################################################################

Review:     Pain, medication use

Number of studies combined: k = 5

                                 g            95%-CI    t p-value
Random effects model (HK-CI) 0.3947 [-0.3345; 1.1239] 1.50  0.2073

Quantifying heterogeneity:
 tau^2 = 0.2561 [0.0250; 2.7751]; tau = 0.5060 [0.1580; 1.6659]
 I^2 = 71.3% [27.3%; 88.7%]; H = 1.87 [1.17; 2.97]

Test of heterogeneity:
     Q d.f. p-value
 13.93    4  0.0075

Details on meta-analytical method:
- Inverse variance method
- Sidik-Jonkman estimator for tau^2
- Q-Profile method for confidence interval of tau^2 and tau
- Hartung-Knapp (HK) adjustment for random effects model (df = 4)
  (with ad hoc correction)

#############################################################################

Review:     Quality of Life

Number of studies combined: k = 18

                                 g            95%-CI    t p-value
Random effects model (HK-CI) 0.0376 [-0.2170; 0.2921] 0.31  0.7593

Quantifying heterogeneity:
 tau^2 = 0.2136 [0.0834; 0.5414]; tau = 0.4621 [0.2888; 0.7358]
 I^2 = 75.6% [61.6%; 84.6%]; H = 2.03 [1.61; 2.54]

Test of heterogeneity:
     Q d.f.  p-value
 69.78   17 < 0.0001
```

```
Details on meta-analytical method:
- Inverse variance method
- Sidik-Jonkman estimator for tau^2
- Q-Profile method for confidence interval of tau^2 and tau
- Hartung-Knapp (HK) adjustment for random effects model (df = 17)
  (with ad hoc correction)
Review:     Sleep

##############################################################################


Number of studies combined: k = 22

                                 g            95%-CI    t p-value
Random effects model (HK-CI) 0.1249 [-0.0673; 0.3171] 1.35  0.1910

Quantifying heterogeneity:
 tau^2 = 0.1472 [0.0602; 0.3351]; tau = 0.3837 [0.2454; 0.5789]
 I^2 = 72.7% [58.2%; 82.1%]; H = 1.91 [1.55; 2.36]

Test of heterogeneity:
     Q d.f.  p-value
 76.79   21 < 0.0001

Details on meta-analytical method:
- Inverse variance method
- Sidik-Jonkman estimator for tau^2
- Q-Profile method for confidence interval of tau^2 and tau
- Hartung-Knapp (HK) adjustment for random effects model (df = 21)
  (with ad hoc correction)

##############################################################################

Review:     Vision

Number of studies combined: k = 36

                                 g            95%-CI    t p-value
Random effects model (HK-CI) 0.0501 [-0.0422; 0.1424] 1.06  0.2876

Quantifying heterogeneity:
 tau^2 = 0.0187; tau = 0.1368; I^2 = 0.0% [0.0%; 37.8%]; H = 1.00 [1.00; 1.27]

Test of heterogeneity:
     Q d.f. p-value
 20.18   35  0.9787

Details on meta-analytical method:
- Inverse variance method
```

```
- Sidik-Jonkman estimator for tau^2
- Hartung-Knapp (HK) adjustment for random effects model (df = NA)
  (with ad hoc correction)
```
