# Supplementary material for: Effectiveness of osteopathic craniosacral techniques: a meta-analysis
Source: Front Med (Lausanne). 2024 Oct 3;11:1452465. doi: 10.3389/fmed.2024.1452465 (PMC11487524; doi:10.3389/fmed.2024.1452465)
Supplement: Supplementary file 3 [file Table_2.PDF]

Question: Craniosacral Therapy compared to sham, standard of care, or no treatment in non-healthy adults

Certainty assessment							№ of patients		Effect		Certainty	Importance
№ of studies	Study design	Risk of bias	Inconsistency	Indirectness	Imprecision	Other considerations	Craniosacral Therapy	sham, standard of care, or no treatment	Relative (95% CI)	Absolute (95% CI)		
**Pain, chronic somatic (assessed with: VAS; Scale from: 0 to 10)**												
8	randomised trials	very serious[a]	very serious[b]	not serious	serious[c]	very strong association	307	301	-	SMD **1.3827 SD higher** (-0.0764 lower to 2.8418 higher)	⊕◯◯◯ Very low	IMPORTANT
**Pain, headache**												
3	randomised trials	serious[d]	not serious	not serious	serious[c]	none	64	66	-	SMD **0.2 SD higher** (-0.47 lower to 0.86 higher)	⊕⊕◯◯ Low	IMPORTANT
**Quality of Life**												
1	randomised trials	very serious[a]	very serious[b]	not serious	serious[c]	none	41	43	-	SMD **0.24 SD lower** (-1.06 lower to 0.59 higher)	⊕◯◯◯ Very low	IMPORTANT
**Disability**												
2	randomised trials	very serious[a]	very serious[b]	not serious	serious[c]	none	49	51	-	SMD **0.05 SD lower** (-1.85 lower to 1.75 higher)	⊕◯◯◯ Very low	IMPORTANT
**Mental Function**												
2	randomised trials	very serious[a]	very serious[b]	not serious	serious[c]	none	31	33	-	SMD **0.33 SD higher** (-1.79 lower to 2.44 higher)	⊕◯◯◯ Very low	NOT IMPORTANT
**Motor Function**												
4	randomised trials	very serious[a]	serious[e]	not serious	serious[c]	none	137	139	-	SMD **0.23 SD lower** (-0.63 lower to 0.17 higher)	⊕◯◯◯ Very low	IMPORTANT

Certainty assessment							№ of patients		Effect		Certainty	Importance
№ of studies	Study design	Risk of bias	Inconsistency	Indirectness	Imprecision	Other considerations	Craniosacral Therapy	sham, standard of care, or no treatment	Relative (95% CI)	Absolute (95% CI)		
**Vision**												
2	randomised trials	very serious[a]	not serious	not serious	serious[c]	none	56	62	-	SMD **0.04 SD higher** (-0.09 lower to 0.16 higher)	⨁◯◯◯ Very low	NOT IMPORTANT

CI: confidence interval; **SMD:** standardised mean difference

Explanations

a. 75% or more of studies ranked as "High Risk of Bias"

b. Subgroup heterogeneity >75%

c. Pooled confidence interval crosses 0

d. 50% to 75% of studies ranked as "High Risk of Bias"

e. Subgroup heterogeneity between 50% and 75%

Supplemental Table 2. Summary of findings in adults and adolescents.
