# Supplementary material for: Effectiveness of osteopathic craniosacral techniques: a meta-analysis
Source: Front Med (Lausanne). 2024 Oct 3;11:1452465. doi: 10.3389/fmed.2024.1452465 (PMC11487524; doi:10.3389/fmed.2024.1452465)
Supplement: Supplementary file 4 [file Table_3.PDF]

Question: Craniosacral Therapy compared to sham, standard of care, or no treatment in non-healthy neonates

Certainty assessment							№ of patients		Effect		Certainty	Importance
№ of studies	Study design	Risk of bias	Inconsistency	Indirectness	Imprecision	Other considerations	Craniosacral Therapy	sham, standard of care, or no treatment	Relative (95% CI)	Absolute (95% CI)		
**Neonate health, behavior**												
6	randomised trials	serious[a]	very serious[b]	not serious	very serious[c]	none	153	152	-	SMD **0.19 SD higher** (-0.37 lower to 0.75 higher)	⨁◯◯◯ Very low	IMPORTANT
**Neonate health, structure**												
2	randomised trials	not serious	very serious[b]	not serious	very serious[c]	strong association	77	79	-	SMD **0.71 SD higher** (-0.11 lower to 1.53 higher)	⨁◯◯◯ Very low	NOT IMPORTANT

CI: confidence interval; **SMD:** standardised mean difference

Explanations

a. >50% of studies rated as "High Risk of Bias"

b. Subgroup heterogeneity >75%

c. Pooled confidence interval crosses 0

Supplemental Table 3. Summary of findings in neonates.
